# NMDA Autoimmune Encephalitis and Severe Persistent Hypokalemia in a Pregnant Woman

**DOI:** 10.3390/brainsci12020221

**Published:** 2022-02-05

**Authors:** Daniela Reisz, Iulia-Gabriela Gramescu, Stefan Mihaicuta, Florina Georgeta Popescu, Doina Georgescu

**Affiliations:** 1 Department of Neurology, “Victor Babeș” University of Medicine and Pharmacy, 300041 Timișoara, Romania; 2 Centrul de Medicina Capului SRL, 300624 Timișoara, Romania; iulia.gramescu@gmail.com; 3 Center for Research and Innovation in Precision Medicine and Pharmacy, Department of Pulmonology, “Victor Babeș” University of Medicine and Pharmacy, 300041 Timișoara, Romania; stefan.mihaicuta@umft.ro; 4 Department of Labor Medicine, “Victor Babeș” University of Medicine and Pharmacy, 300041 Timișoara, Romania; 5 Department of Internal Medicine I, “Victor Babeș” University of Medicine and Pharmacy, 300041 Timișoara, Romania; dgeorgescu@hotmail.com

**Keywords:** anti-N-methyl D-aspartate (NMDA) receptor, NMDAR autoimmune encephalitis, pregnancy, hypokalemia, catatonic state, Gitelman syndrome

## Abstract

**Introduction:** For more than a decade, NMDAR autoimmune encephalitis has been studied and treated as a neurological condition, and good results have been achieve through immune therapies. Apart from being well represented in the CNS, NMDA receptors are currently known of and being studied in multiple non-neuronal cells with potential clinical significance. The association of NMDAR autoimmune encephalitis with pregnancy is rare, and hypokalemia is not mentioned. **Methods:** We present the case of a 30-year-old woman with NMDAR autoimmune encephalitis in her 17th week of pregnancy associated with persistent hypokalemia that had no apparent cause and resisted Kalium chloride supplementation. A diagnostic work-up including clinical, laboratory, and imagistic examinations, was performed. The case was monitored between May and September 2016 at Neurology, SCJUT. **Results:** Severe hypokalemia with normal serum sodium levels persisted throughout the course of clinical manifestation of anti-NMDAR autoimmune encephalitis. **Conclusions**: NMDAR autoimmune encephalitis is under-diagnosed in its atypical clinical variants, and this disease’s association with hypokalemia is not mentioned in the literature. Still, it is of clinical importance because it attests to the implications of other organs/systems in the general autoimmune process of NMDAR encephalitis, and it might change the way we address certain psychiatric disorders by searching underlying organic conditions.

## 1. Introduction

Anti-N-methyl D-aspartate (NMDA) receptor (anti-NMDAR) encephalitis is among the newest neurological clinical entities described in the last few decades. First mentioned in 1997 in association with ovarian teratoma, antibodies to novel neuronal cell membrane antigens were demonstrated later in 2005 (Dalmau and co.) [1]. The NMDA receptors are present mostly in the forebrain and limbic system/hippocampus. They are surface cell antigens (not intracellular as in para-neoplastic encephalitis). This explains the paucity of structural abnormalities in NMDAR autoimmune encephalitis, good recovery with appropriate treatment, and interplay with psychiatric symptoms. Clinical features of NMDAR autoimmune encephalitis may mimic schizophrenia, psychotic spectrum disorders, or substance abuse, and have been well defined in literature since the first cases [2]. Cognitive decline, such as short-term memory deficits, speech problems, movement disorders, alterations of the conscience, epileptic seizures, and autonomic breathing dysregulation develop with time, and some markers are useful for early diagnosis. At least two-thirds of cases have normal MRIs, and one-third have normal CSF [3]. In the case of an adult with progressive psychiatric syndrome and no abnormal neurological parameters, the diagnosis of NMDAR autoimmune encephalitis is easily postponed if the possible red-flag of organicity is overlooked. Recent studies on mice focused on a larger implication of NMDAR in non-CNS responses [4]. These studies have shown the strong implication of kidney, parathyroid, bone, and heart expressing NMDA receptors, with repercussions to the organs’ functions if the receptor is excited or inhibited. The purpose of these studies was to point out the probable relevance to clinical practice. NMDAR autoimmune encephalitis is the most common autoimmune encephalitis. There are around 1000 cases mentioned in the literature [5] to date. In an article published in 2020, Joubert identified a total of 32 cases of pregnant patients diagnosed with concomitant NMDAR autoimmune encephalitis (11 cases identified by his team and 21 in the literature from 2010 to 2019) [6]. From the 11 cases mentioned above, five patients were pregnant during the recovery phase of the NMDAR autoimmune encephalitis. Many atypical forms of NMDAR autoimmune encephalitis are described in the literature (associated with arthritis, chorea [7], meningitis [8], optic neuromyelitis and neurosyphilis [9], herpes simplex virus encephalitis [10], and COVID-19 [11,12,13]). Despite the diversity of the atypical forms mentioned, no severe hypokalemia associated with NMDAR autoimmune encephalitis was described.

## 2. Materials and Methods

We present the case of a pregnant woman with NMDA autoimmune encephalitis, diagnosed and monitored between May and September 2016 in the Neurology I department of the General County Hospital of Timisoara. For the diagnosis, the patient was assessed with multiple brain MRIs, a pelvic MRI, abdominal echography, and CT scans, and radiographic evaluations. Interdisciplinary teams cooperated for the evaluations. The diagnosis was established by lumbar punction and identification of serum and CSF NMDAR antibody (indirect immunofluorescence). Multiple laboratory determinations were made to monitor the kalemic oscillations in correlation with the clinical evolution. Total serum calcium, urinary potassium, urinary calcium, and PTH were determined after an interdisciplinary consultation with the nephrology service at their demand. The tests were done in the hospital laboratory, and normal intervals were found. Informed consent for publication was obtained from the patient, after discharge on a follow-up visit.

## 3. Case Presentation

A 31-year-old pregnant woman was referred by her psychiatrist to the neurology department after ten days of unsuccessful treatment of a depressive episode with psychotic elements. The patient was 17 weeks into pregnancy, her second pregnancy, with a normal gestational course and a normal fetus. Eight years earlier she underwent a depressive episode which was successfully treated. The present illness started with a viral-like episode, dominated by headache, dizziness, unsteadiness, and a slight fever. At that date, no neurological signs were observed. Soon after, the patient got irritated, depressed, agitated, has a suspicious gaze, had anxiety secondarily to possible delusions, became mute, refused to move, and would not eat. The complex neurobehavioral picture of negativism, opposition, stereotyped activities, bizarre feeding, loss of interest for food, and shrinkage of the activity field triggered admission to a psychiatric hospital, where she remained in a catatonic state during the daytime but moved around at night. Mutism precluded the evaluation of abstract thinking and cognitive disorders. Medication (neuroleptics, benzodiazepines, SSRI) was initiated without clinical results, and “resistance to antipsychotic drugs” combined with peculiar clinical aspects, led to admission to the neurology clinic.

At the first neurological examination, the main concern was to assess if the patient’s unresponsiveness was caused by an organic condition or if she was in a catatonic state caused by psychosis. Very few elements of the neurological exam suggested an underlying organic status: polypnea (26/min); tachycardia (111/min). She was in a coma-like state: uncooperative, without spontaneous movements, no movement at the nociception except for grimaces, spontaneous blinking, preserved ocular pursuit movements (spontaneous, unfocused, but not when required), normal pupils, normal photo-motor reflex, no strabismus, resistance to eye-opening. Tendinous reflexes were slightly brisk, with no Babinski sign.

**Figure 1:**
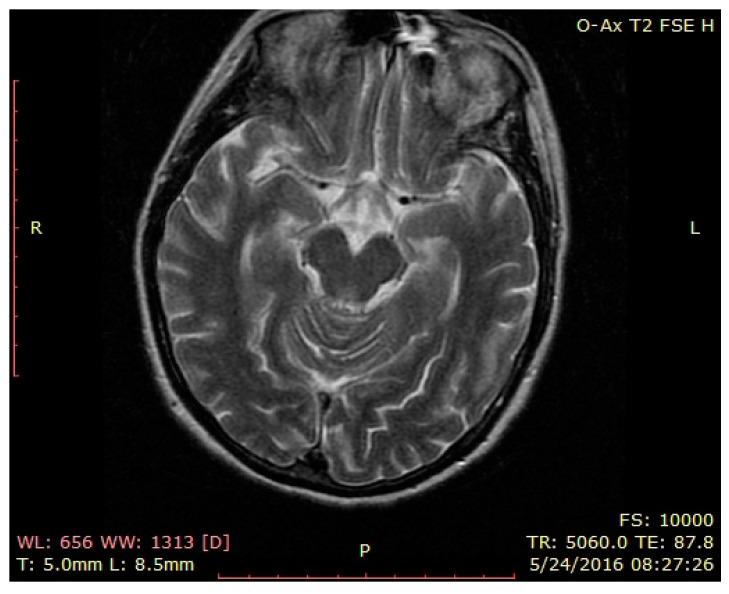
Brain MRI—normal findings (T2 weighted brain scan), axial temporal image.

**Figure 2:**
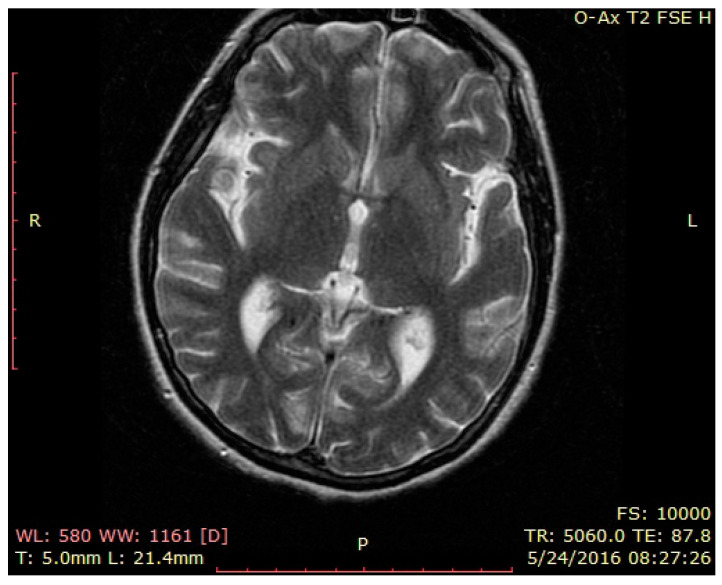
Brain MRI—normal findings (T2 weighted brain scan), axial insular image 1.

**Figure 3:**
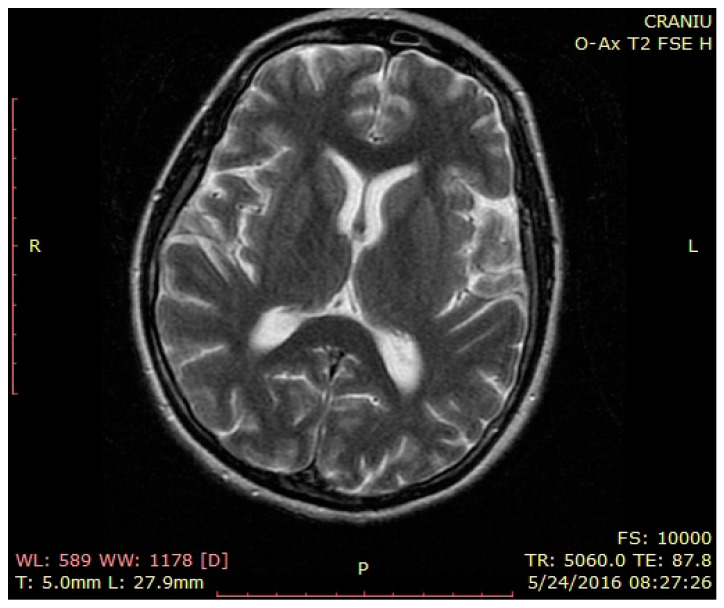
Brain MRI—normal findings (T2 weighted brain scan), axial insular image 2.

Brain MRI—normal findings (Figure 1, Figure 2 and Figure 3—T2 weighted brain scan).

**Figure 4:**
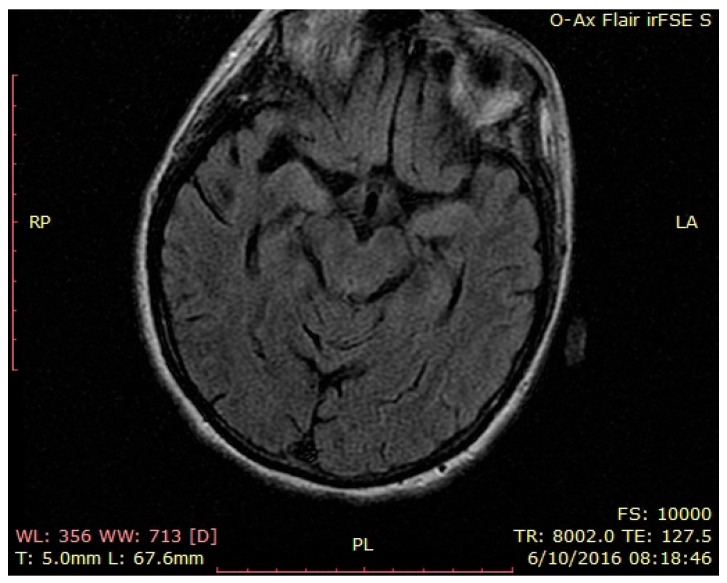
Second brain MRI, FLAIR images, axial temporal image.

**Figure 5:**
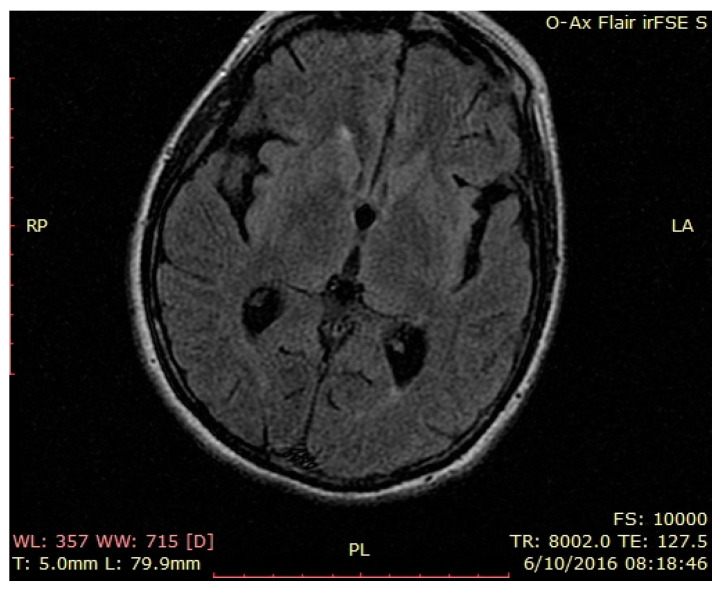
Second brain MRI, FLAIR images, axial insular image.

**Figure 6:**
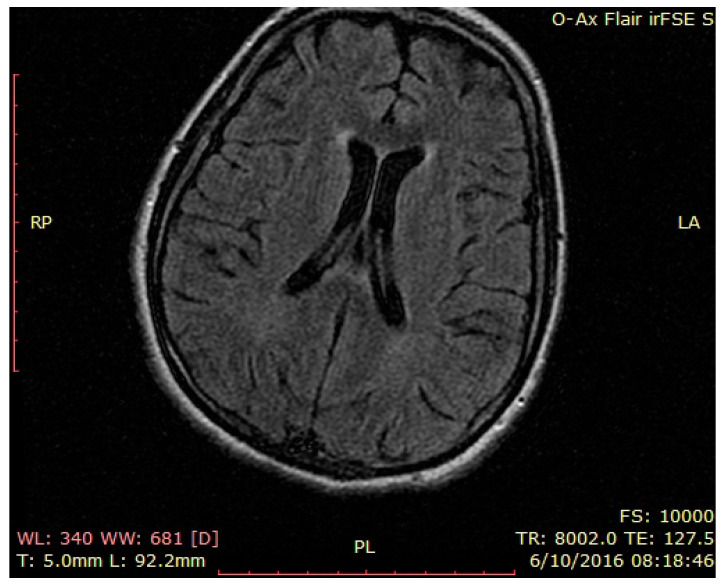
Second brain MRI, FLAIR images, axial parietal image.

Lumbar puncture: cerebrospinal fluid had normal transparency, 5 leucocytes/mL, 15 erythrocytes/mL, 67 mg/mL glucose, 0.49 g/L proteins (normal findings). Laboratory workout was unremarkable: except hypokalemia: 2.3 mmol/L (normal range 3.5–5.1), hypoproteinemia: 5.5 g/dl (normal range 6.4–8.2 g/dl), hypocalcemia: 7.5 mg/dl (normal range 8.5–10.1 mg/dl), blood magnesium normal: 1.8 mg/dl (normal range 1.8–2.4 mg/dl), slight anemia erythrocytes: 3.61 × 10 6/m, thrombocytopenia: 107 × 10 3/m, normal leucocytes: 6.9 × 10 3/m, ESR: 23 mm/1H, CRP: 11.62 mg/L (normal range 0–3 mg/L), inflammatory workout normal, no infection was detected. At the time, we were inclined to consider the entire clinical picture as a catatonic state in a psychotic patient. One of the strong arguments for that idea was the unreactive behavior without physical evidence for neurologic involvement and resistance to eye opening. The clinical picture was completed in the next days with slight myoclonic movements, then an epileptic seizure, followed by coreo-atetotic movements. The patient had periods of opened eyes, spontaneous pursuit gaze, and sporadic pronunciations of the name of her preferred singer. A second brain scan was performed with normal results (Figure 4, Figure 5 and Figure 6—second brain MRI, FLAIR images). Pelvic MRI was normal (Figure S1).

Plasmapheresis began, and the patient showed discreet improvement in neurological status. The hypokalemia remained resistant to the supplementation of daily doses of kalium chloride from 60 mEq KCl/day to 175 mEq KCl + 200 mg Spironolactonum daily or 125 mEq KCl parenteral + 3000 mg KCl oral supplementation + 400 mg Spironolactonum daily. Those large doses of KCl were maintained for months to attain and maintain normal kalemia values.

Table S1 shows the main points of evolution with the corresponding values for seric Potassium and other relevant laboratory findings.

In evolution, she had septicemia as a complication of the central venous catheter, pyothorax with proteus mirabilis and *Staphylococcus aureus*, and miscarriage with spontaneous abortion (Figure S2, and Figure S3 follow-up).

The massive pyothorax imposed thoracocentesis and drainage of the exudate and antibiotics in full doses. In addition, the patient developed hypoparathyroidism with abnormal results of calcium and PTH. The fetal outcome was poor, presumably by septicemia, not by NMDAR autoimmune encephalitis. With the resolution of pyothorax and septicemia, after miscarriage, the patient gradually recuperated conscience, language, and mobility. She was able to cooperate in her recovery process. At follow-up, she had spastic paraparesis, a peculiar childlike voice, acted immaturely and sometimes inappropriately, and she was slightly uncensored (colorful make-up and clothes). As of now, she has no evidence of neoplasia of any kind. After two years, she gave birth to a normal child.

## 4. Discussion

Limbic encephalitis is amongst the newest neurological clinical entities described in the last few decades. Despite the first mention of the NMDAR limbic encephalitis in 1997 as ovarian teratoma-associated limbic encephalitis, insight for a convincing pathogenic proof was offered eight years later by Dalmau and his co-workers in 2005, demonstrating antibodies to novel neuronal cell membrane antigens [1]. In 2007, they reviewed clinical aspects of the disease in a series of twelve women and described epitopes for NMDA receptors. Patients’ antibodies recognize NR1/NR2 heteromers containing the NR2B (and at a lesser degree NR2A) subunit of NMDAR and suggest that the epitopes are conformational [2]. The NMDA receptors are present mostly in the forebrain and limbic system/hippocampus. They are surface cell antigens not intracellular as in other paraneoplastic encephalitides. This explains the paucity of structural abnormalities in NMDA autoimmune encephalitis, good recovery with appropriate treatment, and interplay with psychiatric symptoms.

Few syndromes constantly appear in the evolution of the disease: psychiatric symptoms, cognitive decline as short-term memory deficits, speech problems, movement disorders, alteration of the conscience, epileptic seizure, and autonomic and breathing dysregulation. Sometimes, the disease starts with a transitory fever, headache, or viral infection-like illness [2,3], but this episode could remain unremarkable. In a few weeks, psychiatric or neurological signs arise. If the disease manifests as a psychiatric disturbance, some episodes could be missed. Titulaer and colleagues identified eight classes of symptoms occurring through the timeline of the disease: behavior and cognition, memory, speech, seizures, movement disorder, loss of consciousness, autonomic dysfunction, and central hypoventilation [3]. The disease in adults is more psychiatric (behavioral changes, psychosis), and in children it is more neurological (seizures and movement disorders). At least two-thirds of the cases have normal MRIs (Titulaer series) or half in the Dalmau series, and one-third have normal CSF (79% abnormal CSF in Titulaer series) [3].

The presented case was of a rare variant of autoimmune NMDAR antibody encephalitis in a pregnant woman. The woman had typical features of NMDAR encephalitis: It started with flu-like symptoms. Then depression and irritability developed, followed by neurobehavioral disorders (refusal to eat, to walk, and to speak, but would eat and walk at night). She manifested a bizarre demeanor, alteration of cognitive status, mutism, catatonic-like status, epileptic seizures, choreoathetosis, and autonomic disorders (polypnea), but the confirmation of the diagnosis was late, one month after the onset. Suggestive elements for the diagnosis of NMDAR autoimmune encephalitis develop in time, precluding an early diagnosis. As in Titulaer, her brain MRI was unremarkable, and CSF was normal [3].

Regarding differential diagnosis, a set of hypotheses was prepared. The first one assigned the psychotic presentation to the first psychotic episode of schizophrenia. In the first few days after the presentation in our clinic, a possible catatonic state with a psychiatric cause was discussed. Lejuste notes that resistance to antipsychotic medication is a feature of psychiatric symptoms of NMDAR autoimmune encephalitis [14]. The catatonic state hypothesis was sustained by normal brain and pelvic MRIs, normal laboratory tests, and a normal lumbar punction. Confusion between the two diagnoses is possible because recent findings support an NMDA hypothesis in schizophrenia and mention that “NMDAR hypofunction plays a key role in the pathophysiology of schizophrenia” [15]. Multiple findings support NMDAR hypofunction in schizophrenia. The chemical and immune-mediated blockage of the NMDA receptor produces schizophrenia-like behavior. Genetic mutations in NMDA receptors induce schizophrenic syndrome [15]. 

Due to the normal lumbar punction, we excluded infectious encephalitis (CMV, EBV, HSV, HIV, VZV, and syphilis tested negative). Unavailable tests for the GAD65, VGKC, AMPA, LGI1, Caspr2, and GABA receptor antibodies precluded the possibility to make alternative diagnoses. Repeated attempts to identify an underlying tumor were unsuccessful. 

No obvious cause of hypokalemia was identified. Hypokalemia is rarely associated with pregnancy and was said to have a prevalence of 1% in a large study where underlying causes were identified [16]. We could not find any of those causes in our patient (no gestational hypertension, no pre-eclampsia or eclampsia, hyperemesis gravidarum, no post-partum hemorrhage, no congestive heart failure, no coronary artery disease, no Cushing syndrome, no endogenic or iatrogenic increased insulin levels). Then we supposed hypokalemia to be a part of the autoimmune pathogenic process. 

The association of hypokalemia with hypoparathyroidism raised the suspicion of a more generalized immune process involving not only the brain but other structures (ex., kidney, parathyroide). Bozic and colleagues (2015) discussed the presence of NMDAR in the kidney, bone, parathyroid, and other organs with high involvement in calcium metabolism, and the heart, where transmembranar calcium channels are well represented. There are studies proving the modification of renal function by antagonizing NMDA receptors with consequences at two levels, vasogenic and glomerular [17]. 

Gitelman syndrome is a rare disease, recessively inherited, caused by mutations in the SLC12A3 gene that encodes the thiazide-sensitive sodium-chloride cotransporter [18]; but apart from this, Gitelman-like syndromes exist and case are reported, including in pregnant women [19]. Persistent hypokalemia is a part of the Gitelman syndrome, a rare form of wasting salt nephropathy. A tetrad of symptoms defines the Gitelman syndrome: hypokalemia, metabolic alkalosis, hypomagnesemia, and hypocalciuria [6]. Gitelman syndrome could be causative for seizure disorder in pregnant women, and hypomagnesemia is not present in all cases [20].

In the CNS, NMDA receptors are mostly present in the hippocampal area, modulating brain functions. The existence of NMDA-R or NMDA-like receptors in other organs has recently come to attention. Studies in these areas were conducted only in the laboratory, but a preeminent role of the NMDA receptor in a more general physiological and pathological process outside the CNS has come into focus.

We could stipulate three pathophysiological hypotheses for our case: one type of surface autoantibody directed against NMDA receptors spread throughout various organs, multiple types of autoantibody against multiple organs (kidney, parathyroid gland), and one type of autoantibody acting in the milieu of particular genetics. We could not confirm the pre-existence of the wasting salt nephropathy, nor its persistence after recovery. The wasting salt nephropathy tableau, in this case, seems to have been linked to a single underlying condition. The co-occurrence of pregnancy and NMDAR autoimmune encephalitis is rare, and its association with severe and persistent hypokalemia has not been mentioned elsewhere. The causative link remains to be established.

## 5. Conclusions

NMDAR autoimmune encephalitis is under-diagnosed in its atypical clinical variants. The present study reveals a typical case of NMDA autoimmune encephalitis in its clinical presentation, but atypical in laboratory findings by way of persistent severe hypokalemia. The disease’s associated with hypokalemia is not mentioned in the literature, but it is of clinical importance in multiple respects. The present combination of NMDAR autoimmune encephalitis and hypokalemia is important as a confirmation of previously done preclinical research. NMDAR autoimmune encephalitis is classified as a channelopathy, as is Gitelman syndrome, and this fact reopens this field of research.

There is a possibility that only the kidney or the parathyroid are affected in the NMDAR immune process, and this is to be considered a separated condition despite a unique pathophysiological mechanism.

The article in intended to bring attention the difficulty in managing this variant of NMADR autoimmune encephalitis because the electrolytic imbalance was difficult to control and correct.

1. It attests to the implications of other organs/systems in the general autoimmune process of NMDAR encephalitis.
2. It might change the way we address certain psychiatric diseases by searching for underlying organic conditions.
3. It could permit the diagnosis of a very probable sub-clinical type of NMDAR encephalitis which can be detected not only psychiatrically, but with abnormal laboratory results.

## Data Availability

Data is contained within the article and Supplementary Materials.

## Supplementary Materials

The following are available online at https://www.mdpi.com/article/10.3390/brainsci12020221/s1. Figure S1: Pelvic MRI. Figure S2: Thoracic radiology-pyothorax. Figure S3: Follow up Thoracic radiology. Table S1: Spreadsheet with main clinical and laboratory date.
