# Supplementary material for: NMDA Autoimmune Encephalitis and Severe Persistent Hypokalemia in a Pregnant Woman
Source: Brain Sci. 2022 Feb 5;12(2):221. doi: 10.3390/brainsci12020221 (PMC8869825; doi:10.3390/brainsci12020221)
Supplement: Supplementary file 1 [file brainsci-12-00221-s001.zip › brainsci-1540896-Sup.pdf]

Table S1. Spreadsheet with main clinical and laboratory data.

Day	Symptoms	Seric K (3,5-5,1 mmol/l) Alkaline reserve(22-29 mmol/l) Seric Na (136-145 mmol/L	Total Seric Ca (8,5-10,1 mg/dl) Ca ionic (4,2-5,2 mg/dl)	Urinary K (12-62 mmol/l) Urinary Ca (2-17,5 mg/dl) Urinary Mg (1-13 mg/dl) Urinary Creatinine (30-125 mg/dl) Urinary Na (20-110 mmol/l)	Other laboratory findings	Relevant data	Treatment Management
**1**	Flu-like episode at home, maintain upright position with help because of dizziness. Without objective neurological signs.						
**6**	Agitation, sadness, verbal negativism, verbal stereotypies, diminished activity, fragmentated sleep, diminished appetite, HAM-D=24 (moderate depression) slight vaginal bleeding, normal pregnancy	RA=26,8 mmol/l Na=145 mmol/l	Calcium=8,1 mg/dl		WBC=4,12 10^9/L RBC=3,6.10^9/l Thrombocytes =107 10^9/l		Admission in Psychiatry
**19**	Stuporous, un-cooperant, spontaneous blinking, refuse food, confusion, sleepy, polypnea	K=2,7 mmol/l RA=20 mmol/l Na=144 mmol/L					60 mEq KCl Admission in Neurology
**20**	Stuporous, un-cooperant, Spontaneous blinking, open eyes, eyes pursuit, CGS=5 SO2=92%, Cardiac rhythm=111/min, Respiratory Frequency=26	K=2,8 mmol/L			CSF: glucose=68 (40-70mg/dl) Proteins= 0,29 (0,12-0,60g/l) erythrocytes=15 (0uL), leucocytes=0 (0ul)	Brain MRI-normal findings(Figure 1, Figure2, Figure 3)	Lumbar punction
**22**	Spontaneous open eyes, eyes pursuit	2.3 mmol/L RA=24,7 mmol/l Na=139 mmol/L 2,1	Ca=7,5 mg/dL Ca ionic =3,75mg/dl		Toxicology negative results FT3=3,03 pmol/l, FT4=12,77 pmol/l, TSH=2,718 mIU/ml, BUN=27 mg/dl Mg=1,8mg/dL		60 mEq KCl
**24**	Myoclonic jerks, bedsores	K=2,5 mmol/l Na=138 mmol/l					60 mEq KCl + 50 mg SPL Levetiracetam

**28**	Tonic-clonic jerks, uneven respiratory rhythm, cough.	K=2,1 mmol/l Na=140 mmol/l RA=28,7 mmol/l	Ca=1,05 mmol/l	Ca=17,2 mg/dl Phosphor =33,8mg/dl Na=47 mmol/l K=43,4 mmol/l	Cortisol=24,17 (4,3-22,4 microg/dl, Intact PTH=9,28 (12-65pg/mL) CSF: glucose=67 (40-70mg/dl) Proteins 0,49 (0,12-0,60g/l) erythrocytes=15 (0uL), leucocytes=5(0ul), ALT 122(14-59 U/L), AST=66 (14-37U/L)	Positive Urine Culture-E coli Blood culture-negative Thorax radiography - normal	60 mEq KCl+50 mg SPL Levofloxacin Lumbar punction Gynecologic follow-up
**32**	Right hemi-choreoathetosis, spasmodic torticollis, oppositional contracture, hallucinations, conscient, second MRI normal	K=2,3 mmol/l Na=140 mmol/l	Ca total=8,1 mg/dl Ca=4,14 mg/dl	Ca=8,9 (2-17,5 mg/dl), Phosphor = 3,8mg/dl K=21,6 mmol/l Na=70 mmol/l	BUN=18 (15-45 mg/dl) CR=0,42 (0,57-1,11 mg/dl), ALT=136 (14-59 U/L) AST=78 (15-37 U/L), blood glucose=128 mg/dl		90 mEq KCl Tiapridum 2x100 mg
**35**	Myoclonic jerks	K=2 mmol/l RA=23,1 mmol/l Na=139 mmol/l	Ca=3,58 mg/dl Ca total=7,3 mg/dl		TGP (ALT)=106U/L (14-59 U/L) Mg=1,44 mg/dl (1,8-2,4 mg/dl) BUN=7 (15-39 mg/dl) Cortizol=54,41 (4,3-22,4 microg/dl)	Antibody for NMDAR present.	120 mEq KCl Gynecologic follow-up
**37**	Normal fetus	2,9 mmol/l RA=28 mmol/l Na=137 mmol/l	Ca= 7,3 mg/dl	K =30,1 mmol/l Ca=29,6 mg/dl Mg=2,09 mg/dl Na=212mmol/l	VDRL- negative Anti-HIV-negative AcHBs=9,30 mIU/mL, aHCV=unreactive HAVIgM=0,46S/CO, HBsAg=unreactive, TOXO-IGG<0,5IU/ml, TOXO-IGM=unreactive CMV-IGM=0,338 index, HVG (Virus Herpes Simplex I/II IgG) =41,4 index	Pelvic MRI (Figure S1)	150 mEq KCl + 100 mg SPL Starts plasmapheresis.
**43**	Reacts to sensitive stimuli. Internal strabismus left eye. Facial expressed emotions. Oppositional contracture.	K=2,8 mmol/l RA=28 mmol/l Na=137 mmol/l		Ca=8,5 mg/dl Mg=0,75 mg/dl K=26,9 mmol/l Na=56 mmol/l	TGP (ALT)=34 U/L (14-59 U/L) WBC=5,4 (4-9,5 10^9/L) RBC=3,3 (4-5,5 10^9/L) Platelets=128 (150-400 10^9/L) BUN=6mg/dl	Second MRI-normal (Figure 4, Figure5, Figure 6)	100 mEq KCl + 200 mg SPL+ 2000 mg KCl oral intake
**54**	Fetus normal. Pronounce names. Recognizes husband. She smiles. She has emotional gesture. She says No. Argue mimico-gestual (without words, just	K=4,3 mmol/l RA=25,4 mmol/l Na=138 mmol/l		K=39,7 mmol/l Ca=9,1 mg/dl		Positive Urine Culture-Pseudomonas aeruginosa resistant to Quinolones C-reactive Protein=33,6 (0-3 mg/L)	150 mEq KCl + 400 mg SPL+ 2000 mg KCl oral intake Starts Amikacine Last session of plasmapheresis – allergic reaction

	argumentative prosodie). She tries to uprise.						
**60**	The patient is agitated, has hallucinations, speaks, sings, eats and tries to uprise.	K=4,8 mmol/l RA=26 mmol/l Na=134 mmol/l			BUN=7mg/dl		150 mEq KCl + 400 mg SPL+ 2000 mg KCl oral intake
**64**	Fever=40 gr. Somnolence. Parathyroid insufficiency. Normal fetus.	K=3,6 mmol/l Na=134 mmol/l			WBC=6,7 (4-9,5 10^9/L) RBC=3,32 (4-5,5 10^9/L) Platelets=202 (150-400 10^9/L)		100 mEq KCl + 400 mg SPL
**73**	Spontaneous abortion. Deceased fetus. Somnolence. Apathy	K=4,1 mmol/l	6,1	K =85,9 mmol/l Na=46 mmol/l	WBC=2,1 (4-9,5 10^9/L) RBC=2,87 (4-5,5 10^9/L) Platelets=56 (150-400 10^9/L)		400 mg SPL + 3000 mg KCl oral
**74**		K=2,6 mmol/l			Alkaline reserve= 23 mmol/l (22-29mmol/l)	Thoracic radiology pleral effusion (Figure S2)	125 mEq KCl+400 mg SPL+3000 mg KCl oral
**77**	Severe dyspnea caused by pyothorax with Proteus mirabilis and Staphylococcus aureus. Thoracocentesis. Drenaj	K=1,6 mmol/l			WBC=6,3 (4-9,5 10^9/L) RBC=2,75 (4-5,5 10^9/L) Platelets=96 (150-400 10^9/L) Alkaline reserve= 34,5 mmol/l (22-29mmol/l)	Blood culture - Proteus mirabilis	125 mEq KCl +3000 mg KCL oral + 400 mg SPL Thoracocentesis. Drenaj. Antibiotics
**82**		K=3,7 mmol/l				Pleral effusion culture – Proteus mirabilis, Staphylococcus aureus	125 mEq KCl+400 mg SPL +3000 mg KCl oral intake
**89**	Status epilepticus	K=1,9 mmol/l					125 mEq KCl+400 mg SPL +9000 mg KCl oral intake IGIV, adjusting the AED antibiotics
**97**	Nocturnal agitation. Aggressivity. Depressive mood	K=3,3 mmol/l				Thoracic radiology follow up (Figure S3)	80 mEq KCl+400 mg SPL+3000 mg KCl oral
**104**	The patient speaks, ask for food, wants to uprise.	K=3,6 mmol/l					75 mEq KCL+ 400 mg SPL + oral supplementation The drain tube is stopped.
**118**	The patient is released. She has right hemianopia. She is able to read letter by letter. She is able to calculate. Slow in thinking. Color agnosia.	K=4,2 mmol/l					

**148**	Childlike voice. Puerile behavior. The bizarre actions persists.		Polyclinic evaluation
**248**	Paraparesis, scissor like gait. Brisk osteotendinous jerks. Normal behavior. Acalculia. Good memory for past events and for recent events. Affective indifference.		Polyclinic evaluation
